# Development of an RPA-CRISPR/Cas12a-Based Rapid Diagnosis Strip for the Tangerine Pathotype of *Alternaria alternata*

**DOI:** 10.3390/microorganisms13040826

**Published:** 2025-04-05

**Authors:** Wenge Li, Jintian Tang, Zhiya Ma, Yafen Zhang, Zihong Ye, Huilan Fu

**Affiliations:** 1 Key Laboratory of Microbiological Metrology, Measurement & Bio-Product Quality Security, State Administration for Market Regulation, College of Life Sciences, China Jiliang University, Hangzhou 310018, China; 2 Zhejiang Provincial Key Laboratory of Biometrology and Inspection & Quarantine, College of Life Sciences, China Jiliang University, Hangzhou 310018, China; 3 College of JunCao Science and Ecology, Fujian Agriculture and Forestry University, Fuzhou 350002, China

**Keywords:** Citrus alternaria brown spot, tangerine Pathotype of *Alternaria alternata*, RPA-CRISPR/Cas12a, rapid diagnosis strip

## Abstract

Citrus alternaria brown spot, caused by the tangerine pathotype of *Alternaria alternata*, is one of the most severe fungal diseases affecting citrus crops. Currently, there is a critical need for rapid and visual detection techniques to identify the tangerine pathotype of *A. alternata*. In this study, a novel detection system was developed by combining recombinase polymerase amplification (RPA) with CRISPR/Cas12a technology, targeting the *ACTT3* gene specific to the tangerine pathotype of *A. alternata*. Through optimization of reaction time and component concentrations, the assay demonstrated a detection sensitivity of 1 pg μL^−1^ within 40 min at a constant temperature of 37 °C. The results can be visually interpreted using nucleic acid test strips, offering advantages in specificity, sensitivity, and speed. This system has been successfully validated for the rapid detection of the pathogen within plant tissues, including leaves and fruits, providing an efficient and practical solution for real-time field detection of the tangerine pathotype of *A. alternata*.

## 1. Introduction

Citrus alternaria brown spot is one of the most serious fungal diseases in citrus caused by the tangerine pathotype of *A. alternata*. In China, citrus alternaria brown spot is primarily caused by the tangerine pathotype of *A. alternata*. However, in the United States of America., similar symptoms on oranges and grapefruits are caused by *A. gossypina*, a pathogen not yet reported in China [1]. In the early stages of the disease, small brown spots appear on young leaves and shoot tips. These spots gradually enlarge, often surrounded by a yellow halo, eventually leading to severe defoliation and branch dieback. When *A. alternata* infects the fruit, it forms black, sunken spots on the surface, leading to premature fruit drop [2]. Currently, the management of citrus alternaria brown spot primarily relies on physical removal and chemical control measures [3]. However, there is an urgent need to develop more advanced and rapid detection methods to identify the disease at its early stages. Such methods would enable timely implementation of preventive and control measures, effectively curbing the spread of *A. alternata* and mitigating its impact on citrus crops.

The traditional detection techniques for the tangerine pathotype of *A. alternata* rely on traditional morphological identification and molecular biological methods. While these approaches are widely used, they often face limitations in terms of sensitivity, specificity, and efficiency, highlighting the need for more advanced and rapid diagnostic tools to improve early detection and disease management [4]. The PCR-based diagnostic methods for pathogenic microorganisms are widely utilized in fields including medicine and agriculture. The selection of target sequences in PCR-based diagnostic methods is crucial for their specificity and sensitivity. The host-specific toxins are low-molecular-weight secondary metabolites which are specifically produced by *Alternaria* species or even pathotypes [5]. *Alternaria* citri toxin (ACT) acts as a critical pathogenic factor and is a specific toxin produced only by the tangerine pathotype of *A. alternata* [6]. Thus, some core genes within the ACT biosynthetic cluster exhibit distinct specificity and can be used as specific markers for the molecular detection of the tangerine pathotype of *A. alternata* [7]. A loop-mediated isothermal amplification assay (LAMP) was developed for detection of the tangerine pathotype of *A. alternata* based on the ACT biosynthetic cluster gene *ACTTS2*, which enables the detection of the tangerine pathotype of *A. alternata* within 60 min, with a detection limit reaching 2 pg μL^−1^ [8]. Given the limitations of LAMP technology in primer design complexity, nonspecific amplification, and suboptimal visualization [9,10], there is an urgent need to develop a detection method with stronger result visualization, more user-friendly operation, faster detection speed, and enhanced anti-interference capability and specificity.

Recombinase polymerase amplification (RPA) is a novel nucleic acid amplification technique that enables the isothermal amplification of target double-stranded DNA at a constant temperature range of 37 to 42 °C. It eliminates the need for thermal cycling equipment and can produce billions of DNA copies within 40 min. However, this method is associated with a high rate of false positives [11]. Clustered Regularly Interspaced Short Palindromic Repeats (CRISPR) hold immense potential in the field of nucleic acid detection due to their rapid reaction speed and ease of operation [12]. Particularly, the Cas12a protein, when the specific crRNA recognizes the target DNA sequence, activates its non-specific trans-cleavage activity, cleaving single-stranded DNA containing reporter groups and thereby facilitating signal amplification [13]. This system is characterized by its rapidity, high specificity, and sensitivity. The integration of RPA with CRISPR/Cas12a technology creates a complementary synergy that enhances the overall detection capabilities. On one hand, the amplification capability of RPA can significantly increase the sensitivity of CRISPR/Cas12a detection. On the other hand, the sequence-specific recognition of the CRISPR system can effectively reduce the false-positive results often associated with RPA amplification products [14]. The detection results are displayed using colloidal gold test strips, which provide a stronger visual representation of the outcomes.

In this study, we targeted the *ACTT3* gene within the ACT toxin synthesis cluster of the tangerine pathotype of *A. alternata* to design RPA amplification primers and crRNA for the CRISPR/Cas12a system. By screening various RPA conditions, crRNA cleavage efficiency, sensitivity, and specificity, we ultimately developed a rapid and visual detection system for the tangerine pathotype of *A. alternata*.

## 2. Materials and Methods

### 2.1. The Source of Fungal Strains Used in This Study

The tangerine pathotype of *A. alternata* Z7 was kindly provided by the Institute of Biotechnology, Zhejiang University [4]. The other *Alternaria* fungal strains used in this study, including *A. alternata* f. sp. *mali*, *A. alternata* f. sp. *lycopersici*, *A. brassicicola*, and *A. solani*, were preserved by Zhejiang Provincial Key Laboratory of Biometrology and Inspection & Quarantine. *Botryosphaeria dothidea*, *Colletotrichum karsti*, *Nigrospora lacticolonia*, *Phyllosticta citricarpa*, *Cladosporium tenuissimum*, *Colletotrichum gloeosporioides*, *Malassezia restricta*, *Phyllosticta capitalensis*, and *Pyrenophora teres* f. sp. *teres* were isolated from young citrus leaves exhibiting brown spot symptoms (similar to citrus alternaria brown spot) collected from various trees across multiple orchards. These strains were subsequently identified through a combination of morphological characterization and molecular techniques [15,16].

### 2.2. Detection Target Screening, RPA Primer Design, and crRNA Design

Since the ACT toxin is unique to the tangerine pathotype of *A. alternata*, a key enzyme gene sequence from the ACT toxin synthesis gene cluster, specifically the HMG-CoA hydrolase (ACTT3), was selected as the detection target [17]. PAM sequences (TTTN or NAAA) were searched through *ACTT3* gene (NCBI Accession: AB176852.1) sequence and the crRNAs were designed using CRISPR-P 2.0 (http://cbi.hzau.edu.cn/CRISPR2/ accessed on 10 July 2024) [18]. The PAM sequence-rich region was selected as the target for designing RPA primers. RPA primers, crRNAs, and fluorescent/strips probe were synthesized by Genscript Co., Ltd. (Nanjing, China) (Table 1).

### 2.3. Establishment of the Reaction System

The genomic DNA of fungi and infected samples was extracted using the cetyltrimethyl ammonium bromide method [19].

The RPA amplification kit purchased from Amplification Future Co., Ltd. (Changzhou, China). The amplification reaction was set to a total volume of 50 μL, comprising 29.4 μL of A Buffer, 2 μL each of upstream and downstream primers (10 μmol L^−1^), 5 μL of DNA template, and 9.1 μL of ddH2O added to a dry powder reaction tube. After adding 2.5 μL of B Buffer to the tube cap and mixing thoroughly, the reaction tube was placed at 37 °C for 10–40 min.

EnGen^®^ Lba Cas12a (Cpf1) (New England Biolabs, Co., Ltd., Ipswich, MA, USA) was used in the CRISPR/Cas12a detection system. The reaction mixture included 3 μL of 10 × NEBuffer™ r2.1, an optimized amount of 1 μmol L^−1^ LbaCas12a Nuclease, an optimized amount of 500 nmol L^−1^ crRNA, an optimized amount of 2 μmol L^−1^ ssDNA-FQ/ssDNA-FB, and 1 μL of RPA reaction product, added to 30 μL of nuclease-free water. After thorough mixing, the reaction was performed at 37 °C for 10–30 min, and then we used a real-time fluorescence quantitative PCR instrument (Bio-Rad C1000 Touch™, Hercules, CA, USA) to detect fluorescence intensity (the samples were incubated at 37 °C with signal acquisition performed at 15, 30, 45, 60, 75, and 90 min time points) or using the Cas12/13-specific nucleic acid test strip (Nanjing Warbio Biotechnology Co., Ltd., Nanjing, China) to detect with lateral flow.

### 2.4. Optimization of the RPA-CRISPR/Cas12a Detection System

The cleavage efficiency of the designed crRNAs was evaluated with fluorescence intensity. Each of the three crRNAs was individually incorporated into distinct CRISPR/Cas12 cleavage systems, with water used as the negative control (three repetitions per reaction). The crRNA demonstrating the highest fluorescence intensity within the shortest time frame was identified as having the highest cleavage efficiency.

The optimization of the RPA reaction system primarily focuses on the reaction time and temperature. In the RPA reaction system, the genomic DNA concentration of the tangerine pathotype of *A. alternata* was set at 1 ng. A gradient of reaction times was established at 5, 10, 15, 20, 25, and 30 min, while keeping other reaction conditions constant (three repetitions per reaction). After the reaction, 5 μL of the reaction product was taken for agarose gel electrophoresis to analyze and compare the brightness of the bands. For optimal reaction temperature screening, a gradient of reaction temperatures was set at 33, 35, 37, 39, and 41 °C and screened following the methodology used for screening the reaction time.

### 2.5. Specificity, Sensitivity, and Applications Assay for RPA-CRISPR/Cas12a with the Test Strip Detection

The other *Alternaria* fungal strains and strains isolated from citrus leaves with brown spot symptoms were used for specificity testing. The genomic DNA of each purely cultured strain was extracted with the method described in Section 2.3. Using ddH_2_O as a negative control and *A. alternata* Z7 as a positive control, the specificity of the optimized RPA-CRISPR/Cas12a system was assessed.

The serially diluted genomic DNA of *A. alternata* Z7 at concentrations of 10, 1, 10^−1^, 10^−2^, 10^−3^, and 10^−4^ ng were used as templates of RPA, and the optimized RPA-CRISPR/Cas12a system was used for the sensitivity detection.

To validate the detection efficacy of the RPA-CRISPR/Cas12a system on citrus tissues infected with the fungus, conidia were harvested from 7-day-old *A. alternata* Z7 cultures grown on PDA plates by gentle scraping with sterile ddH_2_O and a conidial suspension was prepared and serially diluted to concentrations of 10^6^, 10^5^, 10^4^, 10^3^, and 10^2^ conidia mL^−1^. One milliliter of each dilution was mixed with 0.2 g of citrus sterile tissue samples (including leaves, stems, and fruits). DNA was extracted using the method described in Section 2.3, and the concentrated DNA was used as a template and was entirely added to the optimized RPA reaction system for the reaction. The detection was performed using DNA from sterile tissue samples as a control.

## 3. Results

### 3.1. crRNA Screening and Cutting Efficiency

**Figure 1:**
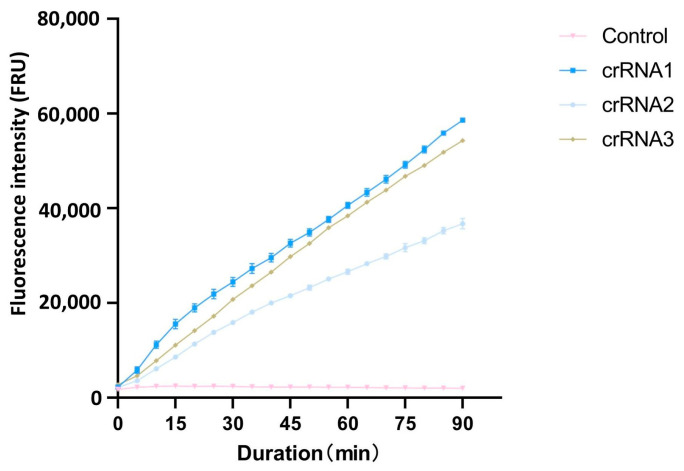
Comparison of different crRNA cutting efficiency.

After PAM sequences analysis with CRISPR-P 2.0, the top three crRNAs with the highest scores were selected as candidate crRNAs for the cleavage efficiency screening (Table 1). Using the full-length cloned *ACTT3* gene as the substrate and ddH_2_O as the control, the cleavage efficiency of the three crRNAs on the target DNA fragment was assessed through the CRISPR/Cas12a fluorescence system. The fluorescence intensity values revealed that, within the same reaction time, the fluorescence intensity in the crRNA1 system was higher compared to that in the crRNA2 and crRNA3 systems. The fluorescence intensity in the ddH_2_O-added control system remained at a low level, indicating that crRNA1 has the highest cleavage efficiency (Figure 1). Therefore, crRNA1 was chosen for subsequent experiments.

### 3.2. Optimization of Reaction Time and Temperature for RPA

**Figure 2:**
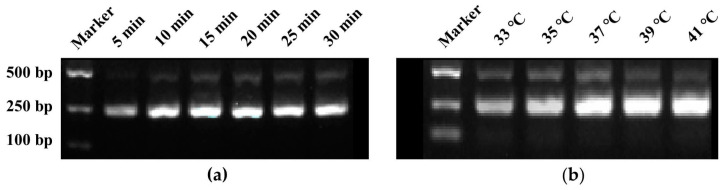
Screening of reaction time and temperature for RPA reaction. (**a**) Reaction time screening, (**b**) Reaction temperature screening.

RPA amplification primers, designated as RPA-F/R (Table 1), targeting the region where crRNA is located were designed, resulting in an amplification product of 241 bp. The agarose gel electrophoresis results of RPA reaction products at different reaction times showed that the brightness of the bands increased with the extension of reaction time. By 15 min, the bands had reached their maximum brightness, indicating that 15 min was the optimal reaction time for the RPA (Figure 2a). Similarly, the electrophoresis results of RPA reaction products at different temperatures revealed that the band brightness gradually increased with the rise in temperature. At 37 °C, the band brightness peaked, thus establishing 37 °C as the optimal temperature for the RPA reaction (Figure 2b).

### 3.3. Optimization of the Ratio of Cas12a to crRNA, the Concentration of Cas12a and crRNA, and the Concentration of ssDNA in the CRISPR/Cas12a Reaction System

**Figure 3:**
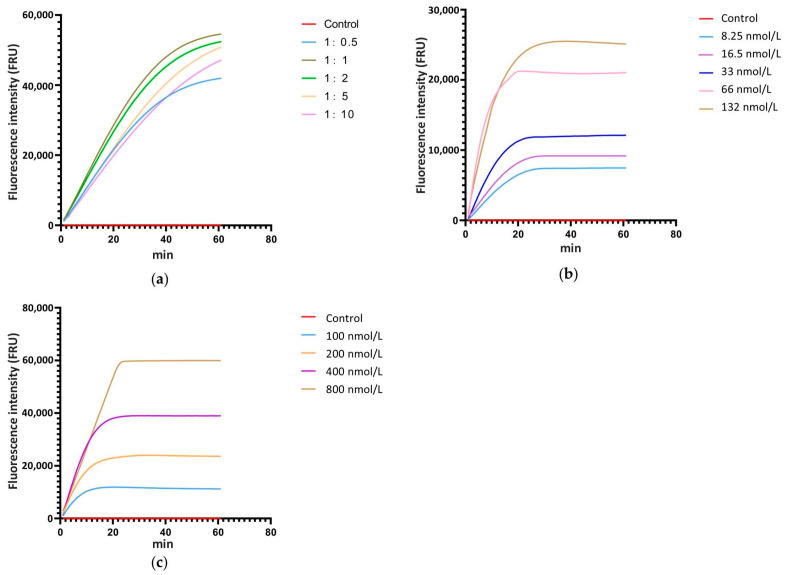
Screening of the ratio ofCas12a to crRNA, the concentration of Cas12a and crRNA, and the concentration of ssDNA in the reaction system with a real-time fluorescence detection. (**a**) The ratio of Cas12a to crRNA, (**b**) the concentration of Cas12a and crRNA, (**c**) the concentration of ssDNA.

The ratio of Cas12a to crRNA, the concentration of Cas12a and crRNA, and the concentration of ssDNA are critical factors that significantly influence the cleavage efficiency of the CRISPR/Cas12a system. Thus, real-time fluorescence detection with ssDNA-FQ was used for system optimization. According to the relevant studies, the concentration of Cas12a and ssDNA-FQ in the reaction system was set at 33 nmol L^−1^ and 200 nmol L^−1^, respectively, and the concentration ratios of Cas12a to crRNA were established at 1:0.5, 1:1, 1:2, 1:5, and 1:10, with ddH_2_O as the control. The real-time monitoring of fluorescence values for each reaction showed that when the concentration ratio of Cas12a to crRNA was 1:1, the fluorescence intensity at the same time point was the highest compared to systems with other ratios (Figure 3a). Therefore, the optimal concentration ratio of Cas12a to crRNA is 1:1. Following the 1:1 concentration ratio of Cas12a to crRNA, a gradient of Cas12a and crRNA concentrations was set at 8.25 nmol L^−1^, 16.5 nmol L^−1^, 33 nmol L^−1^, 66 nmol L^−1^, and 132 nmol L^−1^, with ddH_2_O as the control and 200 nmol L^−1^ of ssDNA-FQ. Real-time monitoring of fluorescence values for each reaction was conducted. The results showed that as the concentration increased, the fluorescence intensity at each time point also increased, with the peak fluorescence intensity rising and the time required to reach the peak decreasing (Figure 3b). Considering factors such as fluorescence intensity at various time points, the time required to reach peak fluorescence intensity, and the economic cost of the reaction, 33 nmol L^−1^ was selected as the optimal concentration for Cas12a and crRNA. According to the screened reaction condition with 33 nmol L^−1^ Cas12a and 33 nmol L^−1^ crRNA, a gradient of ssDNA-FQ concentrations was set at 100 nmol L^−1^, 200 nmol L^−1^, 400 nmol L^−1^, and 800 nmol L^−1^, with water as the control. Real-time monitoring of fluorescence values for each reaction system was conducted. The results indicated that as the concentration of ssDNA-FQ in the system increased, the fluorescence intensity at each time point also increased and the peak fluorescence intensity rose. When the ssDNA-FQ concentration was at 400 nmol L^−1^ and 800 nmol L^−1^, the fluorescence values at each time point were similar within the first 20 min of the reaction, showing a significant enhancement compared to concentrations of 100 nmol L^−1^ and 200 nmol L^−1^ (Figure 3c). Therefore, 400 nmol L^−1^ was chosen as the optimal concentration.

### 3.4. Screening of Reaction Time for CRISPR/Cas12a with the Test Strip Detection

**Figure 4:**
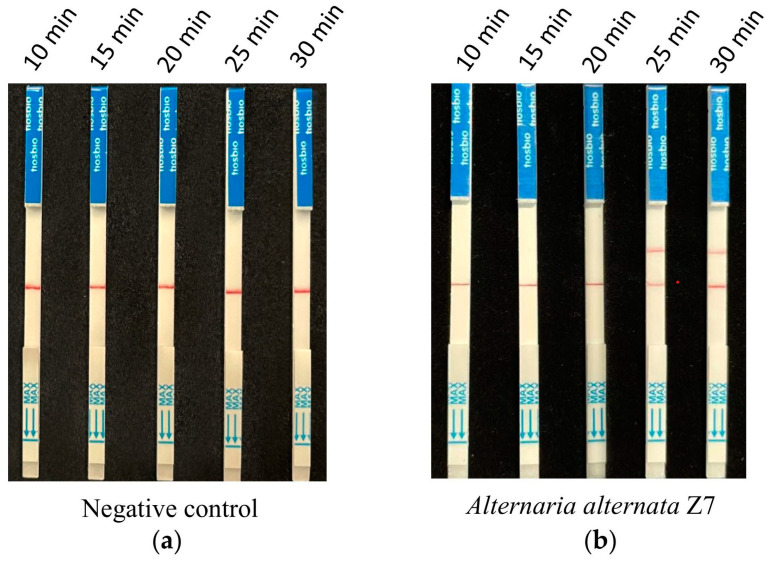
The reaction time for CRISPR/Cas12a with the strip detection. (**a**) Negative control with ddH_2_O as RPA template, (**b**) experimental group with *A. alternata* Z7 genomic DNA as RPA template.

According to the optimized reaction system of RPA and CRISPR/Cas12a screened from the previous assays, 1 ng of *A. alternata* Z7 genomic DNA was used for the RPA reaction, with ddH_2_O serving as the negative control. One microliter of the RPA reaction product was utilized for the CRISPR/Cas12a reaction, where ssDNA-FB replaced ssDNA-FQ. Gradient reaction times of 10, 15, 20, 25, and 30 min were set for the CRISPR/Cas12a system, and the products from different reaction times were subjected to test strip detection. The results indicated that in the control reactions, all detection outcomes exhibited a band only at the control line (Figure 4a). In the reaction groups with *A. alternata* Z7 genomic DNA as the template, all detection results showed a band at the control line, and bands at the test sample line appeared at reaction times of 25 and 30 min. Consequently, the CRISPR/Cas12a reaction time for the test strip detection was set at 25 min (Figure 4b).

### 3.5. Specificity and Sensitivity Testing of the RPA-CRISPR/Cas12a Test Strip Detection System

**Figure 5:**
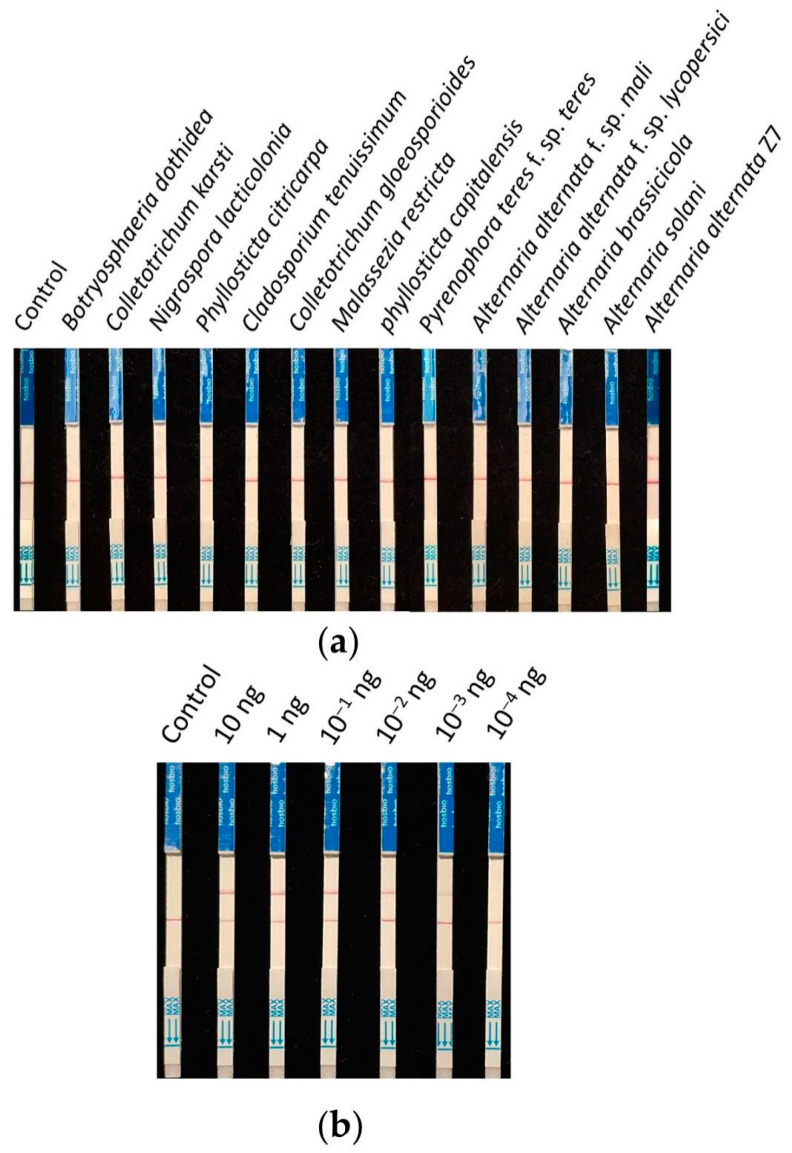
Specificity and sensitivity testing of the RPA-CRISPR/Cas12a test strip detection system. (**a**) Specificity testing, (**b**) sensitivity testing.

Nine strains (isolated from young citrus leaves exhibiting brown spot symptoms) and four strains of *Alternaria* (two of which were other pathotypes of *A. alternata*) were collected for specificity testing. The detection was carried out according to the previously screened system and reaction conditions, using ddH_2_O as the control. The results showed that all control lines had bands, and only the *A. alternata* Z7 strain exhibited a test sample band (Figure 5a). Therefore, it is demonstrated that this detection method can specifically identify the tangerine pathotype of *A. alternata*.

The genomic DNA of *A. alternata* Z7 at the quantity of 10, 1, 10^−1^, 10^−2^, 10^−3^, and 10^−4^ ng was used as templates, with ddH_2_O as the control, and added to the RPA reaction system, respectively. One microliter of the RPA reaction product was subjected to the CRISPR/Cas12a reaction and then detected using test strips. The results indicated that in systems with templates ranging from 10 ng to 10^−3^ ng DNA, the test sample bands were visible, whereas in the system with 10^−4^ ng DNA as the template, no sample bands could be detected (Figure 5b). This demonstrates that the sensitivity of the RPA-CRISPR/Cas12a detection system is 10^−3^ ng (1 pg) genomic DNA.

### 3.6. Applications Assay for RPA-CRISPR/Cas12a with the Test Strip Detection

**Figure 6:**
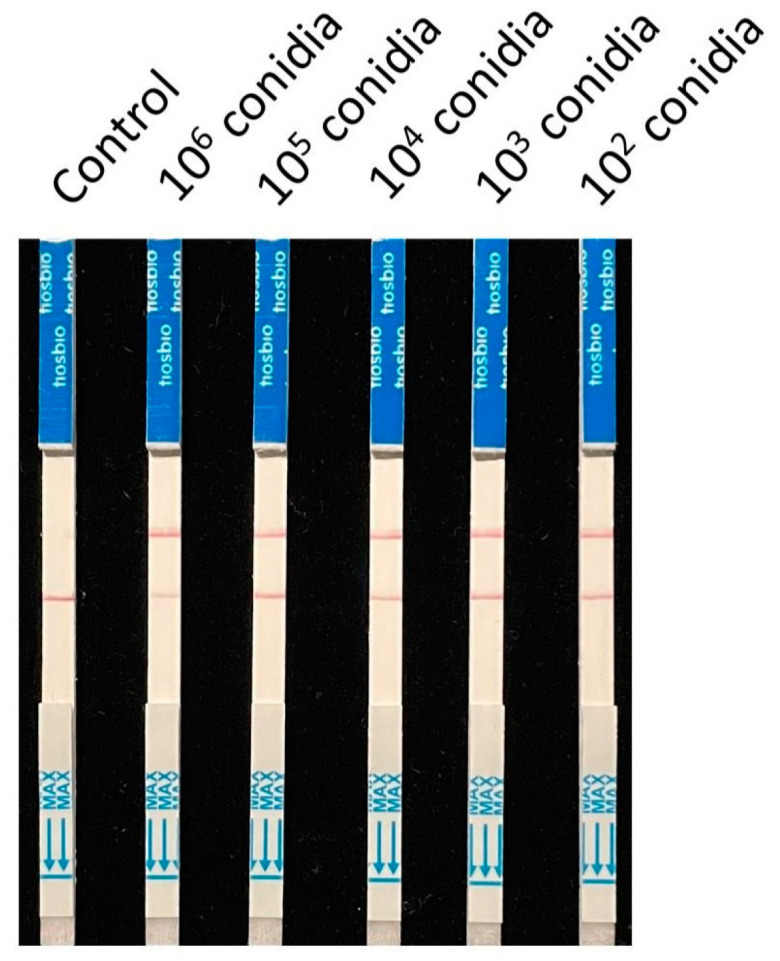
Detection of mixed samples of citrus tissue and *A. alternata* Z7 conidia.

To validate the application of this RPA-CRISPR/Cas12a detection system in the field, 10^6^, 10^5^, 10^4^, 10^3^, and 10^2^ conidia of *A. alternata* Z7 were mixed with 0.2 g of sterile citrus tissue, respectively, and the total DNA was extracted for detection. The DNA from sterile tissue samples was set as a control. The results showed that *A alternata* Z7 could be detected in all mixed samples, proving that the detection method developed in this study based on the RPA-CRISPR/Cas12a test strip detection system is suitable for the detection of the Citrus alternaria brown spot pathogen, the tangerine pathotype of *A. alternata*, in the field (Figure 6).

## 4. Discussion

Many pathogenic fungi can infect citrus leaves and fruits, resulting in dark spot-like lesions. Among these, the Citrus alternaria brown spot caused by the tangerine pathotype of *A. alternata* is one of the most common. Due to the high similarity of symptoms caused by various pathogens in their early stages, it is challenging to distinguish them. Therefore, there is an urgent need to develop corresponding detection methods. However, there are seven different pathotypes of *A. alternata* based on the host specificity [7]. Consequently, it is difficult to differentiate based on the morphological characteristics of the pathogens and some traditional molecular detection targets such as ITS, EF-α, and the like. Given the specificity of host-specific toxins among different pathotypes of *A. alternata*, the key synthesis genes of these toxins were selected as specific molecular detection targets for detection with traditional PCR and LAMP [7]. We conducted a comparative analysis of the ACT toxin synthesis gene cluster in the genomes of different strains of the tangerine pathotype of *A. alternata*, confirming the conservation of the *ACTT3* gene sequence. Therefore, in subsequent experiments, there is no need to verify the conservation of the target in different strains of the tangerine pathotype of *A. alternata* with RPA-CRISPR/Cas12a.

Currently, RPA-CRISPR/Cas12a technology has been recognized as a new generation of rapid nucleic acid detection technology due to its convenience, speed, and sensitivity, and it has good application prospects in the field of rapid detection of pathogens [14]. The RPA-CRISPR/Cas12a-based test strip detection technology for the tangerine pathotype of *A. alternata* developed in this study enables the visualization of 1 pg of genomic DNA from the tangerine pathotype of *A. alternata* in the reaction system within 40 min. Compared to the traditional PCR and LAMP detection technologies developed in earlier research [7], this method offers faster speed and more convenient visualization.

To more closely approximate the actual application scenarios in the field, the strains used for specificity detection in this study were isolated from pathogens resembling Citrus alternaria brown spot lesions or non-pathogenic bacteria around the lesions. For application validation, a mixture of citrus tissue and conidia of the tangerine pathotype of *A. alternata* was used for extraction, effectively simulating field scenario detection. The results also indicated that the detection sensitivity of this method for conidia can reach 100 per reaction system.

In summary, this study, targeting the key ACT toxin synthesis gene *ACTT3*, is the first to develop a rapid, visual detection strip for the tangerine pathotype of *A. alternata* based on RPA-CRISPR/Cas12a. It offers improvements in detection sensitivity, detection time, and the visualization of detection results compared to traditional PCR and LAMP methods.

## Figures and Tables

**Table 1:** Primers, crRNAs, and probe sequences used in this study.

Experiment	Primer Name	Sequence (5′-3′)
RPA amplification	RPA-F	CCAAGGCGAAGCGATGTTAAAGTCTAGCACA
	RPA-R	CATACCCTATCAGTCGCGGGAGATAGAAGCT
crRNA for CRISPR/Cas12a	crRNA-1	UAAUUUCUACUAAGUGUAGAUCUUUUGUUAGAAGAGGAAUC
	crRNA-2	UAAUUUCUACUAAGUGUAGAUCCACUGGCGCCUUGUAUCCG
	crRNA-3	UAAUUUCUACUAAGUGUAGAUUAAAGCGAAGUGGCAAUACU
Fluorescent detection probe for CRISPR/Cas12a	ssDNA-FQ	FAM-/TTATTATT/-BHQ1
Lateral flow (strips) detection probe for CRISPR/Cas12a	ssDNA-FB	FAM-/TTATTATT/-Biotin

## Data Availability

The original contributions presented in this study are included in the article. Further inquiries can be directed to the corresponding authors.
